# Advances in Chromodomain Helicase DNA-Binding (CHD) Proteins Regulating Stem Cell Differentiation and Human Diseases

**DOI:** 10.3389/fcell.2021.710203

**Published:** 2021-09-20

**Authors:** Caojie Liu, Ning Kang, Yuchen Guo, Ping Gong

**Affiliations:** State Key Laboratory of Oral Diseases, West China School of Stomatology, Sichuan University, Chengdu, China

**Keywords:** epigenetic, chromodomain helicase DNA-binding protein, chromatin remodeling, histone modification, CHARGE syndrome

## Abstract

**Background:** Regulation of gene expression is critical for stem cell differentiation, tissue development, and human health maintenance. Recently, epigenetic modifications of histone and chromatin remodeling have been verified as key controllers of gene expression and human diseases.

**Objective:** In this study, we review the role of chromodomain helicase DNA-binding (CHD) proteins in stem cell differentiation, cell fate decision, and several known human developmental disorders and cancers.

**Conclusion:** CHD proteins play a crucial role in stem cell differentiation and human diseases.

## Introduction of CHD Superfamily

There are three classes of epigenetic modifier proteins, including chromatin writers, erasers, and readers. We center around a chromatin reader superfamily and review their functions and structures as well as known roles in human diseases. In eukaryotic organisms, ATP-dependent chromatin remodeling enzymes are commonly divided into three major superfamilies, including SWItch/sucrose non-fermentable (SWI/SNF), Imitation SWI (ISWI), and chromodomain helicase DNA-binding (CHD) proteins (Dann et al., 2017; Barisic et al., 2019).

CHD comprises nine proteins, which are classified as three subfamilies on account of domain homology. These nine CHDs all involve tandem chromatin organization modifier domains (chromodomain) as well as sucrose non-fermentable2 (SNF2)-like ATP-dependent helicase domains (Flanagan et al., 2005; Bajpai et al., 2010; He et al., 2016; Farnung et al., 2017). Recently, a study reviewed the structure of these four domains and the differences between CHDs (Mills, 2017; Goodman and Bonni, 2019). CHDs read and/or interpret histone modifications by specialized domains. When reading the chromatin state, CHDs disrupt the DNA–histone interaction via translocating the nucleosomes along the same or the other DNA strand (Clapier et al., 2017; Mashtalir et al., 2018).

**FIGURE 1:**
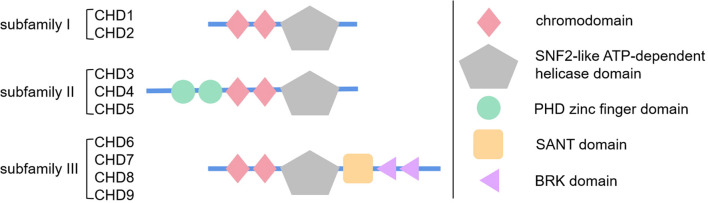
Schematic diagram showing the structure and specific domains of three subfamilies of chromodomain helicase DNA-binding (CHD) proteins. This figure briefly demonstrates the basic structure and domains of CHD protein family. As is reviewed in section “Introduction of CHD Superfamily” of this article, CHD proteins are mainly divided in three subfamilies by their specific domains, subfamily I with basic chromodomian and sucrose non-fermentable2 (SNF2)-like ATP-dependent helicase domain, subfamily II with additional PHD zinc finger domain, and subfamily III with additional SANT domain and BRK domain. The simple geometric patterns represent the protein domains, whose size or location are not strictly to scale, just for brief demonstration.

CHDs share highly similar helicase-ATPase domains with the SWItch2/SNF2 superfamily (Noh et al., 2015; Mashtalir et al., 2018). These helicase-ATPase domains provide energy. At the same time, they promote disruption of histone-DNA contacts as mentioned above (Clapier et al., 2017; Mashtalir et al., 2018). Moreover, the three subfamilies described above are defined by specific domains (Figure 1). Briefly, subfamily I, including CHD1 and 2, share DNA-binding domains that demonstrate similar function with SWI3, ADA2. Subfamily II, including CHD3-5, contain the PHD zinc finger domain in addition to the three domains mentioned above, which promotes its binding to methylated histone residues and protein cofactors. Subfamily III, including CHD6-9, its specific SANT domain could promote non-specific DNA binding (Mills, 2017; Platt et al., 2017).

Chromodomains were first recognized in *Drosophila* heterochromatin protein 1. Heterochromatin protein 1 owns a chromodomain that binds to nucleosomes to facilitate a closed chromatin state as well as regulate homeotic genes (Flanagan et al., 2005; Farnung et al., 2017; Zhao et al., 2020). It is known nowadays that binding to methylated histone residues is the primary function of chromodomains. CHDs contain a special variant of the chromodomains with methy1-binding cages, which promote interactions with H3K4me (Flanagan et al., 2005; Dann et al., 2017; Barisic et al., 2019). For instance, CHD1 chromodomains interact with H3K4me, and CHD5 chromodomains bind to H3K27me3 (Dorighi and Tamkun, 2013; Link et al., 2018; Goodman and Bonni, 2019). Therefore, CHDs demonstrate special functions and preferences for active or repressive histone marks. CHD chromodomains are essential for proper gene expression and maintaining dynamic chromatin structures.

## CHD and Stem Cells

**FIGURE 2:**
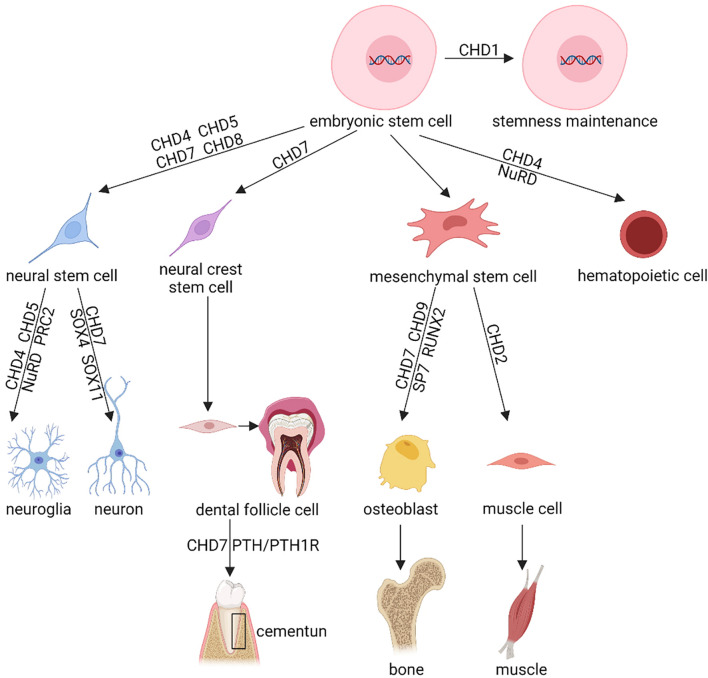
Schematic diagram showing the regulatory effects of CHD proteins in stem cell proliferation, differentiation, and functioning. This figure briefly demonstrates the known function of the CHD protein family in various stem cells, including the stemness maintenance, cellular differentiation, lineage commitment, cell fate decision, etc. Shown are representative stem cells, e.g., Embryonic Stem Cells (ESCs), Neural Stem Cells (NSCs), neural crest stem cells, Mesenchymal Stem Cells (MSCs), reviewed in section “CHD and Stem Cells” of this article. This diagram only exhibits the certified function of CHD protein family, the uncertain or controversial effects of CHDs have been omitted. NuRD, nucleosome remodeling and deacetylase; PRC2, Polycomb Repressive Complex 2.

CHD superfamily proteins are essential to regulate gene expression. Thus, CHDs are crucial for the survival, maintenance, and proliferation of stem cells as well as regulating the cell fate of their daughter cells (Figure 2).

### Embryonic Stem Cells (ESCs)

ESCs demonstrate an open chromatin environment. Via activation or repression of different genetic pathways, ESCs differentiate toward mesenchymal, hematopoietic, neural, and other lineage cells. By maintaining open chromatin, CHD1 participates in the mediator complex, a regulator of ESCs (Gaspar-Maia et al., 2009; Bulut-Karslioglu et al., 2018). This complex is a multiprotein complex that pre-initiates gene transcription through binding to CHD1 and recruiting it to express genes (de Dieuleveult et al., 2016; Percharde et al., 2017). If lacking CHD1, chromatin would condense to form heterochromatin. In that case, due to increased ectodermal lineage gene expression and decreased endodermal lineage gene expression, pluripotent differentiation would be impaired (Lin et al., 2011; Koh et al., 2015; Suzuki et al., 2015). Furthermore, induction of *CHD1* is necessary for efficient programming of induced pluripotent stem cells (de Dieuleveult et al., 2016; Percharde et al., 2017; Bulut-Karslioglu et al., 2018). Therefore, CHD1 is essential to maintain pluripotency in stem cells.

By regulating epigenetic and signaling pathways, CHD7 is also crucial for cell fate decisions. In mouse ESCs, the *Chd7* gene is highly expressed and associated with gene expression active signals. In stem and progenitor cells, the euchromatic chromatin environment is poised between activation and repression (Yang et al., 2017). Different from active or inactive states, those promoters as well as enhancers typically express both active and inactive marks (Schnetz et al., 2009; Yang et al., 2017). Via binding to active as well as poised enhancers of ectodermal lineage genes, CHD7 plays an important role in histone modifying, transcription factor recruiting, and other chromatin remodeling (Platt et al., 2017; Goodman and Bonni, 2019). Meanwhile, it promotes open chromatin at enhancers of critical genes such as *Sox2, Nanog*, and *Oct4* (Engelen et al., 2011; Puc and Rosenfeld, 2011; Fujita et al., 2016).

In brief, the CHD family is essential to regulating the function of ESCs.

### Neural Stem Cells (NSCs)

NSCs produce both neuron and supporting cells and, thus, play a pivotal role in the nervous system as well as sensory organs. In the dentate gyrus of the hippocampus and subventricular zone of the forebrain, CHD4, CHD5, and CHD7 cooperate with signaling pathways and transcription factors, which are critical for the differentiation and function of NSC niches (Feng et al., 2017; Weiss et al., 2020; Parenti et al., 2021).

Binding to the polycomb repressive complex 2 (PRC2) during cortical neurogenesis, CHD4 was found to express in the murine subventricular zone neural progenitor (Mohd-Sarip et al., 2017; Pierson et al., 2019; Shieh et al., 2020). This complex represses the expression of glial fibrillary acidic protein gene (*Gfap)* as well as blocks glial differentiation (Dorighi and Tamkun, 2013; Sparmann et al., 2013). During neurogenesis, this complex promotes neuronal differentiation by inhibiting the *Gfap* locus (Sparmann et al., 2013). Moreover, CHD4, together with other nucleosome remodeling and deacetylase (NuRD) complex, can repress several genes that downregulate neuronal differentiation (Mohd-Sarip et al., 2017; Link et al., 2018; Pierson et al., 2019; Shieh et al., 2020).

CHD5 also binds to PRC2 and H3K27me3 in NSCs (Egan et al., 2013; Xie et al., 2015; Hayashi et al., 2016). In the subventricular and subgranular zones in the hippocampus, high expression of CHD5 is found in neuroblast cells as well as neural progenitors (Egan et al., 2013). CHD5 is pivotal for learning and memory. It is also found that depletion of *Chd5* in the developing cortex leads to reduction of migratory neuroblasts (Egan et al., 2013).

CHD7 is also vital for fine function of NSCs. CHD7 is highly expressed in both the subgranular and subventricular zones in adult mice. In these areas, CHD7 colocalizes with markers of NSCs, neural progenitor cells, and neuroblasts (Feng et al., 2017; Goodman and Bonni, 2019). Studies in which *Chd7* was conditionally deleted in the adult subventricular zone demonstrate that CHD7 deletion resulted in a reduction of mature dopaminergic NSCs (Feng et al., 2013). Such *Chd7* deficiency also downregulates the expression of proneural genes, such as *Sox4* and *Sox11* (Feng et al., 2013; Brajadenta et al., 2019). Conditional knockout of *Chd7* in the subgranular zone also reduces neurogenesis (Feng et al., 2013). Furthermore, in the otic placodes and olfactory, CHD7 promotes NSC progenitor proliferation (Jones et al., 2015; Ohta et al., 2016; Whittaker et al., 2017). CHD7 is critical for NSC function although the mechanisms by which CHD7 regulates NSC function remain to be determined.

### Mesenchymal Stem Cells (MSCs)

As a kind of multipotent mesoderm-derived cell differentiating into myoblasts, adipocytes, osteoblasts, and chondrocytes, MSCs are shown to be regulated by CHD proteins (Mohd-Sarip et al., 2017). Several different CHD proteins regulate the differentiation of MSCs into four distinct lineages. CHD2 is critical for induction of myogenic cell fates (Harada et al., 2012; de Dieuleveult et al., 2016; Semba et al., 2017; Nieto-Estevez and Hsieh, 2018). CHD9 could bind to osteocalcin, which is one of the master transcriptional factors for bone development, and promote its expression (Shur et al., 2006a,b; de Dieuleveult et al., 2016). Recently, we found that CHD7 is essential for osteogenic differentiation of human MSCs. Depletion of *CHD7* via siRNA impairs the osteogenesis potential of MSCs, and overexpression of *CHD7* via lentivirus vector could promote the osteogenesis potential of MSCs. Mechanically, we found that CHD7 might bind to the enhancer of *Sp7*, and it also interacts with SMAD1, indicating that CHD7 is crucial to the osteogenesis potential of MSCs (Chen et al., 2016).

Besides the above-described biological processes, the CHD family also affect the development and functional maintenance of many organ systems, such as the hematopoietic and circulatory systems (Koh et al., 2015; Sperlazza et al., 2015; Zhen et al., 2017; Arends et al., 2019; Hsu et al., 2020; Tu et al., 2021). For example, CHD7 promotes the osteogenesis potential of dental follicle cells to form cementum by upregulating the PTH/PTH1R signaling pathway (Liu et al., 2020).

## CHD and Human Diseases

### CHD in Neurodevelopmental Disorders (NDDs)

Due to the important regulatory role of the CHD family in the differentiation and function of NSCs, the deletion or mutation of CHD genes often leads to NDDs, featuring as intellectual disability (ID), autism spectrum disorders (ASDs), and epilepsy.

Several *CHD*, such as *CHD2*, *CHD6*, *CHD7*, and *CHD8*, were found non-sense, heterozygous, or other kinds of mutations in patients with ASD, ID, and epilepsy (Allen et al., 2013; Suls et al., 2013; Sugathan et al., 2014; Ellingford et al., 2021; Parenti et al., 2021). In patients with ASD and/or ID that associate with gastrointestinal disturbance and macrocephaly, 13 recurrent alleles of *CHD8* were figured out (Bernier et al., 2014; Sugathan et al., 2014; Ellingford et al., 2021). Using RNA- and ChIP-sequencing, a recent study indicates that knockdown of *CHD8* could not alter the neural ectodermal or morphology markers of neural progenitors, but could impair their gene expression (Sugathan et al., 2014; Goodman and Bonni, 2019). CHD8 was found to bind CHD7 as well as p53, regulate p53, and inhibit cell proapoptotic effects during development (Nishiyama et al., 2009; Hurley et al., 2021; Tu et al., 2021). Interestingly, CHD7 could also bind to and repress p53 (Van Nostrand et al., 2014; Corsten-Janssen and Scambler, 2017). Thus, some CHD proteins might share common target genes, interacting factors, and downstream mechanisms. CHD2 mutations were also observed in patients with epilepsy (Allen et al., 2013; Suls et al., 2013; Nieto-Estevez and Hsieh, 2018). All these data raise the possibility that the region of *CHD* mutation may be wider than the previous hypothesis. Due to the intricacy of CHD targets as well as associating partners, a main goal of future studies is making clear the mechanisms by which *CHD* mutation disturbs NSCs and neuronal development.

### CHD in CHARGE Syndrome and Kallmann Syndrome

CHARGE is the acronym of an autosomal dominant genetic syndrome, which is characterized by six main symptoms, including ocular coloboma, congenital heart defects, choanal atresia, developmental retardation, genital anomalies, and ear anomalies (Pagon et al., 1981; Brajadenta et al., 2019). According to a Canadian study, this syndrome occurs in approximately 1 in 10,000 live births (Issekutz et al., 2005; Van Nostrand et al., 2014). *CHD7* has been closely linked to this disorder because heterozygous mutations in this gene were found in more than 90% of these patients (Vissers et al., 2004; Ghaoui et al., 2015; Brajadenta et al., 2019). Therefore, CHD7 is also one of the most researched members among the CHD superfamily. The embryologic expression of CHD7 involves several sites, including eyes, olfactory bulb cells, inner ears, etc. (Vissers et al., 2004; Jamadagni et al., 2021). Additionally, high expression of *CHD7* is also observed in undifferentiated neuroepithelium and neural crest mesenchyme as mentioned above. Thus, CHARGE is a monogenic disorder with variable expressivity (Jones et al., 2015; Yan et al., 2020). However, due to the multiplicity of *CHD7* mutations as well as the variable expressivity of this syndrome, no critical genotype/phenotype correlations can be found (Bajpai et al., 2010; Butcher et al., 2017). In an *in vitro* study, intact recombinant CHD7 protein was purified and proved to be an ATP-dependent nucleosome remodeling factor (Bouazoune and Kingston, 2012; Yan et al., 2020). Interestingly, when CHARGE patients were administrated with recombinant CHD7 protein, their enzymatic activity, which is related to chromatin remodeling, reduced in a mutation-specific mode (Rother et al., 2020; Yan et al., 2020). This study supports the hypothesis that CHD7 haploinsufficiency is the main cause of CHARGE syndrome (Whittaker et al., 2017; Brajadenta et al., 2019). Although other functions of CHD7 should be discovered, these functions might help explain the clinical features in CHARGE patients.

*CHD7* haploinsufficiency leads to dysfunction in sensory processes as well as impaired vision, hearing, balance, and olfaction. A mouse model has been established and analyzed to learn more about the role of CHD7 in CHARGE. *Chd7* knockout mice embryos cannot survive over embryonic day 10.5, but heterozygous mice show several similar defects observed in CHARGE syndrome (Kim et al., 2008; Gage et al., 2015; Jones et al., 2015). Moreover, there is no CHARGE syndrome patient who is figured out to have *CHD7* homozygous mutations (Vissers et al., 2004; Basson and van Ravenswaaij-Arts, 2015). These facts suggest that homozygous mutations in *CHD7* might cause embryonic lethality, possibly due to the wide expression of the *CHD7* gene in tissues affected in CHARGE syndrome (Yan et al., 2020). Using conditional *Chd7*^*flox*^ allele mating with tissue-specific *Cre* transgenes, a recent study found that CHD7 is necessary for eye development in multiple embryonic tissues and also essential for lens development in the surface ectoderm (Gage et al., 2015; Goodman and Bonni, 2019). *Chd7*^+/–^ mice showed hypoplasia and aplasia of posterior and lateral semicircular canals as well as innervation defects of the vestibular sensory epithelium (Yan et al., 2020; Jamadagni et al., 2021). In all, this evidence suggests that CHD7 might play a similar role in sensory tissues as in NSCs.

As two major phenotypes observed in CHARGE syndrome patients, hyposmia and anosmia mean decrease and loss of the smell sense, respectively (Brajadenta et al., 2019; Yan et al., 2020). Olfactory deficiency is usually accompanied by aplasia or hypoplasia of the olfactory bulbs (Ghaoui et al., 2015). Through behavioral assays and electrophysiological study, *Chd7* heterozygous mice were found lacking in odor discrimination, olfactory bulb hypoplasia, and complete anosmia (Layman et al., 2009). As an essential gene in stem cell differentiation, high expression of CHD7 could be found in olfactory NSCs as well as progenitor cells (Whittaker et al., 2017; Jamadagni et al., 2021). Depletion of *Chd7* results in a significant reduction of NSC proliferation in olfactory epithelials, thus leading to a decrease in olfactory receptor neurons and delayed recovery post damage (Feng et al., 2017; Whittaker et al., 2017). Moreover, efferent neurons in the olfactory epithelium as well as olfactory bulb neurogenesis from the subventricular NSC niches of such mutant mice are impaired, resulting in the reduction of tyrosine hydroxylase-positive interneurons in the olfactory bulb (Ohta et al., 2016; Feng et al., 2017). Taken together, olfactory processing counts on CHD7 function.

Through co-IP and chromatin immunoprecipitation studies, CHD7 was proved to interact with *SOX2*, which associated with several diseases, such as Feingold syndrome, Alagille syndrome, and Pallister–Hall syndrome (Engelen et al., 2011; Puc and Rosenfeld, 2011; Fujita et al., 2016). These syndromes share several similar phenotypes with CHARGE syndrome, including tracheoesophageal defects, genital abnormalities, semicircular canal hypoplasia, and pituitary and endocrine dysfunction (Puc and Rosenfeld, 2011; Stamou et al., 2020). It is reported that CHD7 binds to SOX2 because of the massive overlap in expression as well as function of these two proteins. Besides this, CHD7 and SOX2 share similar functions in the development of ectodermal lineages that are influenced in CHARGE syndrome (Schnetz et al., 2010; Doi et al., 2017). As one of the characters of CHARGE syndrome, craniofacial malformations are also commonly observed (Van Nostrand et al., 2014; He et al., 2016). It was found that *Chd7* is necessary for proper craniofacial development via immunofluorescence and *Cre* lineage tracing (Sperry et al., 2014). Importantly, CHD7 also interacts with SMAD1 and could bind to the enhancer region of *Sp7*, which is a master transcription factor of osteogenic differentiation (Chen et al., 2016).

Besides this, CHD7 is also closely associated with Kallmann syndrome, which is a genetic heterogeneous congenital disease mainly characterized by idiopathic hypogonadotrophic hypogonadism (IHH) (Kim et al., 2008; Stamou et al., 2020). Such manifestation is largely caused by impaired gonadotropin-releasing hormone (GnRH) (Balasubramanian and Crowley, 2017; Stamou et al., 2020). The pathogenic mechanism of Kallmann syndrome is complicated and could be partially explained by several mutated genes, including the missence mutation of *CHD7*, leading to the alteration of the domain or function of CHD7 protein (Kim et al., 2008; Boehm et al., 2015). Some of the clinical symptoms of Kallmann syndrome, e.g., anosmia, IHH, heart defect, cleft lip, cleft palate, etc., also often appear in CHARGE syndrome, so these two syndromes are often compared in clinical work and need differential diagnosis (Ufartes et al., 2018; Stamou et al., 2020).

### CHD in Cancers

CHD proteins are also involved in cancers. Genomic and epigenomic changes are correlated and could predict the tumor phenotypes and progression.

CHD1 was proved to be frequently deleted in prostate cancer (Attard et al., 2016; Zhang et al., 2020). Researchers conducted and analyzed whole-genome, whole-transcriptome, and DNA methylation data from patients with primary prostate cancer and healthy controls. Deletions in CHD1 occurred in 18% of the tumors (Li et al., 2020). Further studies confirm that CHD1 plays a key role in myeloid-derived suppressor cell recruitment and find that CHD1/IL6 is a major regulator of the immunosuppressive tumor microenvironment in prostate cancer (Zhao et al., 2020).

CHD4 is closely associated with breast, endometrial, and colorectal cancer (Novillo et al., 2021). As a crucial ingredient in NuRD complex, the upstream regulating effects of CHD4 involves the recruitment of DNA methyl transferase and key transcriptional repressors (Hata et al., 2019; Wang et al., 2020). CHD4 could recruit inhibitory chromatin remodelers to the DNA damage repair sites and initiate and support the silencing of tumor suppressor gene. Such functions confirm the oncogenic effect of CHD4 (Chang et al., 2019; Novillo et al., 2021). In addition, CHD4, as a co-activator of hypoxia-inducible factor (HIF), is upregulated in human breast tumors and is related to the expression of HIF target genes (Shieh et al., 2020; Wang et al., 2020). Besides this, CHD4 was associated with poorer overall survival in breast cancer patients (Novillo et al., 2021).

According to the position, expression pattern, and function of CHD5 in neuroblastoma cells and xenograft cells, CHD5 was identified as a tumor suppressor gene (Kolla et al., 2014; Liu et al., 2018). CHD5 also functions as one of the tumor suppressor genes in other types of tumors, e.g., gliomas, breast, colon, lung, ovarian, and prostate cancers (Bagchi et al., 2007; Xie et al., 2015). Especially, low expression of CHD5 is strongly associated with poorer clinical and biological characteristics and prognosis (Kolla et al., 2014; Higashi et al., 2015).

To sum up, all of the associations among these diseases emphasize the variety of cell processes that require proper chromatin remodeling.

## Conclusion

Here, we review the CHD superfamily as well as known functions in several kinds of stem cells. The CHD family is widely involved in and regulates many physiological and biochemical pathways in the organism, which is of great significance for normal growth, development, and functional maintenance of the body as well as the occurrence and development of several diseases. Future studies aiming at revealing the proper mechanisms by which CHDs mediate these effects will uncover more important cues about this important chromatin remodeler.

## Author Contributions

CL: drafting manuscript. PG: approval of article and funding secured. YG: literature summary, article revision, and funding secured. NK: concept, design, and funding secured. All authors contributed to the article and approved the submitted version.

## Conflict of Interest

The authors declare that the research was conducted in the absence of any commercial or financial relationships that could be construed as a potential conflict of interest.

## Publisher’s Note

All claims expressed in this article are solely those of the authors and do not necessarily represent those of their affiliated organizations, or those of the publisher, the editors and the reviewers. Any product that may be evaluated in this article, or claim that may be made by its manufacturer, is not guaranteed or endorsed by the publisher.
